# Spatial-Temporal Variation of Bacterial Communities in Sediments in Lake Chaohu, a Large, Shallow Eutrophic Lake in China

**DOI:** 10.3390/ijerph16203966

**Published:** 2019-10-17

**Authors:** Lei Zhang, Yu Cheng, Guang Gao, Jiahu Jiang

**Affiliations:** 1 School of Civil Engineering and Architecture, Chuzhou University, Chuzhou 239000, China; 17856000876@163.com; 2 State Key Laboratory of Lake Science and Environment, Nanjing Institute of Geography and Limnology, Chinese Academy of Sciences, Nanjing 210008, China; guanggao@niglas.ac.cn (G.G.); jiangjh@niglas.ac.cn (J.J.)

**Keywords:** Microbial community, Functional genus, LEfSe analysis, Illumina high-throughput sequencing, Co-occurrence patterns

## Abstract

Sediment bacterial communities are critical for the circulation of nutrients in lake ecosystems. However, the bacterial community function and co-occurrence models of lakes have not been studied in depth. In this study, we observed significant seasonal changes and non-significant spatial changes in the beta diversity and community structure of sediment bacteria in Lake Chaohu. Through linear discriminant analysis effect size (LEfSe), we observed that certain taxa (from phylum to genus) have consistent enrichment between seasons. The sudden appearance of a Firmicutes population in spring samples from the Zhaohe River, an estuary of Lake Chaohu, and the dominance of Firmicutes populations in other regions suggested that exogenous pollution and environmental induction strongly impacted the assembly of bacterial communities in the sediments. Several taxa that serve as intermediate centers in Co-occurrence network analysis (i.e., Pedosphaeraceae, Phycisphaeraceae, Anaerolineaceae, and Geobacteraceae) may play an important role in sediments. Furthermore, compared with previous studies of plants and animals, the results of our study suggest that various organisms, including microorganisms, are resistant to environmental changes and/or exogenous invasions, allowing them to maintain their community structure.

## 1. Introduction

Lakes are critical components of ecosystems that couple biogeochemical cycles and play an irreplaceable role in the daily lives of humans [1]. At present, due to urbanization and irregular land-use patterns, a large amount of external pollution enters lakes, especially in developing countries [2,3,4,5,6]. Many lines of evidence suggest that such pollution affects freshwater quality and, more importantly, worsens the trophic level of freshwater lakes [7,8,9].

Fortunately, recent studies have found that nutrient exchange between water and sediments plays a key role in regulating the trophic levels of water in aquatic ecosystems [10], and it is well documented that these exchanges, such as the transformation and biogeochemical cycling of nitrogen and phosphorus, are driven by sediment bacteria [8,11]. In addition, shifts in microbial community diversity and structure can be induced by various physicochemical properties, including pH [12], Dissolved oxygen (DO) [13], nutrients [14] (nitrogen, phosphorus) and other contaminants (such as polycyclic aromatic hydrocarbons). Hence, understanding the composition of bacterial communities in sediments can provide important insights into the environmental changes in local ecosystems [15,16]. However, understanding only community composition does not provide insights into complex microbial community structure and interactions between microorganisms and makes it more difficult to predict the impact of the environment on community composition.

Network analysis tools and strategies have been used by scholars to explore interactions between entities at both the macro- [17,18,19] and micro-levels [20,21,22,23]. Network analysis can explain the structural properties and associations between nodes [24,25]. Furthermore, co-occurrence network analysis of complex microbial community structures provides insight into the co-occurrence patterns between communities [26,27]. Liang et al. (2017) studied the co-occurrence model of soil bacterial communities during vegetation restoration [28]. Jia et al. (2016) studied the co-occurrence model of bacterial communities in petroleum-contaminated soils [29]. Delafont et al. (2016) studied the environmental factors of free-living amoeba and related bacterial communities through a network of drinking water [30]. To date, microbial co-occurrence networks have been preliminarily explored, but such initial explorations of this type of co-occurrence model have not established a robust association between microorganisms, as the sample sets should include as many temporal and spatial gradients as possible to determine if there is sufficient variability to resolve the co-occurrence patterns [26]. Therefore, a deep understanding of the symbiotic models of bacterial communities is still lacking. However, understanding only the diversity of bacterial communities and co-occurrence patterns does not fully capture the diversity that exists within the system. Functional predictions can reproduce the main findings of the microbial analysis and facilitate metagenomic predictions in a wide range of host-related and environmental samples [31].

We focus on the benthic microbial community of a large eutrophic lake to determine seasonal and spatial variabilities in sample microbial communities. Our aim is to answer the following three questions. (i) Will the diversity and composition of bacterial communities in shallow eutrophic lake sediments change due to seasonal and regional changes? (ii) Does the effect of seasonal changes on the composition and diversity of the bacterial community exceed that of regional changes? (iii) What is the bacterial function of sediments in eutrophic shallow lakes affected by nutrients?

## 2. Materials and Methods

### 2.1. Study Site Description and Sampling Procedure

**Figure 1:**
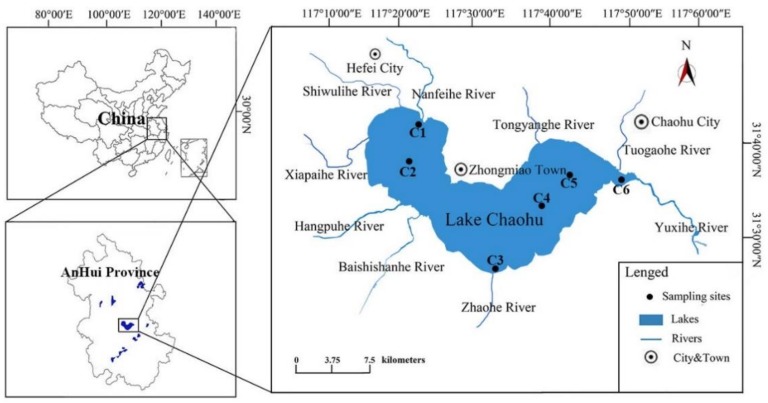
Location of sampling sites in the eutrophic Lake Chaohu, Anhui, China.

Located in the middle of Anhui Province, China, Lake Chaohu (30°25′28″–31°43′28″N, 117°16′54″–117°54′46″E) is the fifth largest freshwater lake and one of the most eutrophic lakes in China [32]. There are 33 tributaries of Lake Chaohu, and the Nanfei River, Zhao River and Yuxi River are the main tributaries [33,34] (Figure 1). Since the 1970s, Lake Chaohu has suffered from severe eutrophication [35,36,37]. Comprehensive research on pollution in Lake Chaohu is ongoing, and many mitigation measures have been implemented [38,39]. However, lake eutrophication continues [40], and the release of endogenous nutrient loads from sediments may be one of the main reasons [41].

To summarize the overall situation of the lake, we selected three sampling points: the estuary of the Nanfei River (C1: 31°41′26″N, 117°24′1″E), the Yuxi River (C6: 31°35′33″N, 117°48′13″E) and the Zhaohe River (C3: 31°26′3″N, 117°33′4″E). C2 (31°37′59″N, 117°22′43″E) and C5 (31°35′38″N, 117°41′43″E) were located in the center of East Lake and West Lake, respectively. C4 (31°31′14″N, 117°37′38″E) was located in the Lake Chaohu source of drinking water. The average sedimentation rate of the 210 Pb dating method in Lake Chaohu was 0.25 cm/a [42]. We collected 24 samples from the six sites in four seasons (summer: August 2016, autumn: November 2016, winter: February 2017, and spring: May 2017). Sample name was abbreviated. For example, CSM1 to CSM6 represent samples of sediments from C1 to C6 at the Lake Chaohu sampling site in May, where C represents Lake Chaohu, S represents sediment, and M represents May.

Surface sediment samples were taken with a Peterson sampler, and three parallel samples were taken at each site and mixed into one sample. DO (dissolved oxygen), pH and T (temperature) were measured immediately after sample collection. The sediment was placed in a sterile polyethylene bottle and shipped on ice to the laboratory, where it was immediately centrifuged at 6000 rpm for 10 min. The extracted water (i.e., concentrate) was collected. After measuring TP (total phosphorus), TN (total nitrogen) and TOC (total organic carbon), the sediment was extracted through a 0.45-μm cellulose acetate membrane and partially dehydrated. Finally, the dehydrated sediment was stored at −26 °C for DNA extraction, 16S rRNA gene polymerase chain reaction (PCR) amplification and Illumina MiSeq sequencing.

### 2.2. Chemical Analysis

DO, pH and T were measured after sample collection using a multiparameter controller (YSI 6600V2, Yellow Springs, OH, USA). The TOC, TN and TP in the sediments were measured in the laboratory according to standard methods [43].

### 2.3. DNA Extraction and Polymerase Chain Reaction (PCR) Amplification

DNA was extracted from an aliquot of 0.25 g of sediment from each sample using an MP Biomedicals Fast DNA^TM^ SPIN Kit according to the manufacturer’s instructions, and the genomic DNA concentration and purity were detected by an ultraviolet spectrophotometer (Eppendorf, Germany).

The V3-V4 region of the bacterial 16S ribosomal RNA gene was amplified by PCR (94 °C for 5 min, followed by 25 cycles at 94 °C for 30 s, 55 °C for 45 s, and 72 °C for 1 min and a final extension at 72 °C for 5 min) using the barcoded primers 515F (5′-GTGCCAGCMGCCGCGGTAA-3′) and 907R (5′-CCGTCAATTCMTTTRAGTTT-3′). Subsequently, PCR products were detected by 1.5% agarose gel electrophoresis. The PCR products were purified using AMPure Beads and finally sent to Personal Biotechnology Co., Ltd., Shanghai, China, for sequencing.

### 2.4. Sequencing Data Optimization and Operational Taxonomic Units (OTU) Clustering Analysis

We merged the paired-end 16S reads, trimmed primers and distal bases and removed quality-filtered sequences and singletons using USEARCH [44]. The RDP Classifier (version 2.2, http://sourceforge.net/projects/rdp-classifier/) Bayesian algorithm (confidence threshold of 0.7) was used to obtain the species classification information corresponding to each OTU, and OTU-representative sequences were classified at a similarity level of 97% using the QIIME platform [45] (http://qiime.org/scripts/assign_taxonomy.html). The raw sequencing data have been submitted to the NCBI Sequence Read Archive under project accession code SRP216986.

PICRUSt (Phylogenetic Investigation of Communities by Reconstruction of Unobserved States) is a tool for predicting the metabolic function of microbes [31]. PICRUSt compares 16S rRNA gene sequence data with the Greengenes database, searches for the “reference sequence nearest neighbors” for each sequence and obtains functional annotations from the KEGG (Kyoto Encyclopedia of Genes and Genomes) database [46] based on the taxonomic unit. We reconstructed the potential functional gene families in each sample community. The second level included a total of 45 metabolic pathway subfunctions, the third level corresponded to the metabolic pathway map, and the fourth level corresponded to the specific annotation information for each KO (KEGG Orthology) group or cluster in the metabolic pathway.

### 2.5. Statistical Analyses

We visualized the Bray–Curtis distance matrix using NMDS (non-metric multidimensional scaling). Analysis of similarity (ANOSIM) statistics were calculated to test for significant differences between the prior sampling groups based on the environmental parameters. First, the distance between two samples was calculated using the Bray–Curtis algorithm. The R value was calculated and then recalculated after sample replacement. NMDS and ANOSIM statistics were obtained using R-3.5.3 with the vegan package [47]. All statistical analyses excluded OTUs with an abundance below 0.01%. Furthermore, one-way ANOVA (analysis of variance) was performed using SPSS version 19.0. Results with P < 0.05 between groups were declared statistically significant.

A Co-occurrence network model of the bacterial taxonomic groups was built by selecting the top 50 by abundance, and if the Spearman correlation coefficient had a P value between 0.01 and 0.6, it was considered statistically robust [26]. We used the igraph package to calculate the number of nodes, the number of edges, and the minimum path length [48]. RDA (redundancy analysis) was employed to eliminate redundant variables based on other measured variables, automatically select variables with large effects, and gradually eliminate redundant parameters according to the values of the variance inflation factor. Moreover, LDA (linear discriminant analysis) and LEfSe (LDA effect size) analysis were conducted to search for biomarkers that were significantly different between groups [49]. Finally, partial RDA was used to compare community KO profiles between the sampling locations and seasons.

## 3. Results

### 3.1. Alpha Diversity

The microbial community diversities and phylogenetic structures of twenty-four samples were analyzed by Illumina high-throughput sequencing. A total of 502,008 bacterial sequences were identified in this study after the removal of low-quality sequences and chimeras. The high-quality sequences obtained were classified as having a similarity of 0.97. The rarefaction curves indicated (Figure S1) that the sediment bacterial OTUs obtained at the current sequencing depth were sufficient to represent the bacterial community. Moreover, Good’s coverage values of the sample illustrated that the original Lake Chaohu microbes were correctly represented by the bacterial profiles. Furthermore, the total number of OTUs varied among the 24 samples (Table S1). The total number of OTUs was between 1259 and 1644 in spring and between 1364 and 1616 in fall; thus, the numbers were similar between these two seasons. However, in summer (from 1419 to 1685) and winter (from 1427 to 1673), the number of OTUs showed a significant increase (Table S1).

The Shannon index, which emphasizes the richness component of diversity [50], ranged from 9.65 to 9.90 and 9.26 to 9.91 in summer and winter, respectively, but from 8.84 to 9.50 and 8.99 to 9.68 in autumn and spring, respectively. Therefore, the diversities in summer and winter were higher than those in autumn and spring. The inverse Simpson index represents the evenness component of diversity [50]. The results indicated that the evenness components of diversity were similar among the seasons and sites (Table S1).

### 3.2. Beta Diversity

**Figure 2:**
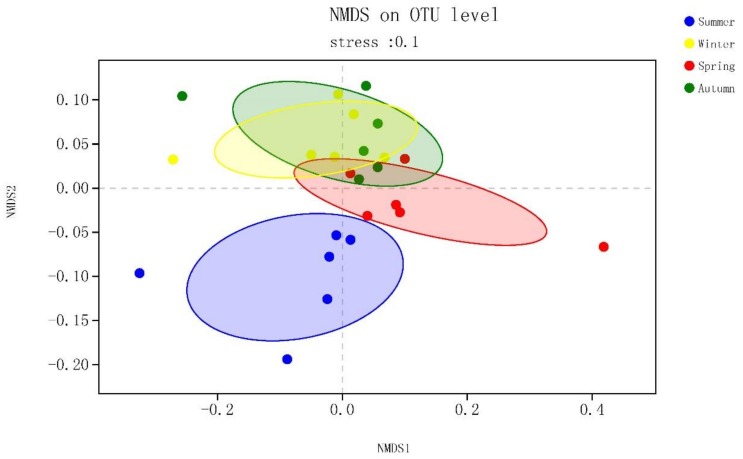
Non-metric multidimensional scaling diagram showing bacterial composition differences obtained among the 24 sampling sites.

A Bray–Curtis distance matrix was analyzed with NMDS to explore the beta diversity of all samples. The horizontal and vertical coordinates of the NMDS plot indicate relative distances and have no practical significance. The bacterial communities of the sediments displayed distinct seasonal groups (Figure 2). In contrast, the NMDS plot demonstrated very inconspicuous spatial groups. The consistency of the results was verified using Bray–Curtis dissimilarities based on ANOSIM statistics (Table 1). Season (r = 0.319, P = 0.001) had a more significant effect on the sediment bacterial community than space (r = −0.083, P = 0.929).

### 3.3. The Spatiotemporal Distribution of Bacterial Communities

**Figure 3:**
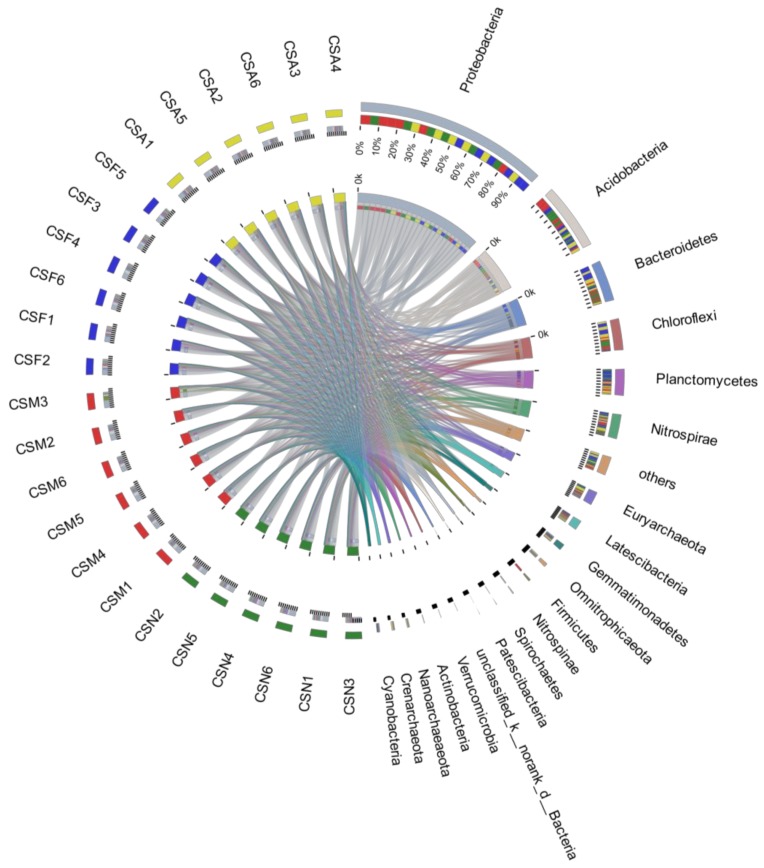
Distribution of the microbial community for each sample at the phylum level. Circos [51] visualized the data. The width of the bars for each phylum indicates the relative abundance of the phylum in the sample. CS indicates the sediments of Lake Chaohu, A (August), F (February), M (May) and N (November) indicate four months, and 1 to 6 indicate sampling points C1 to C6.

Spatiotemporal changes in bacterial communities can be assessed by directly comparing the relative abundances of taxa or by the LEfSe algorithm. Direct comparisons (Figure 3) revealed that the bacterial community was dominated by Proteobacteria (~39.87%), specifically Deltaproteobacteria (~13.50%), Betaproteobacteria (~13.56%), Gammaproteobacteria (~11.20%) and Alphaproteobacteria (~1.44%). These taxa were followed by Acidobacteria (~13.19%), Chloroflexi (~6.68%), Bacteroidetes (~6.10%) and Planctomycetes (~5.88%). 

**Figure 4:**
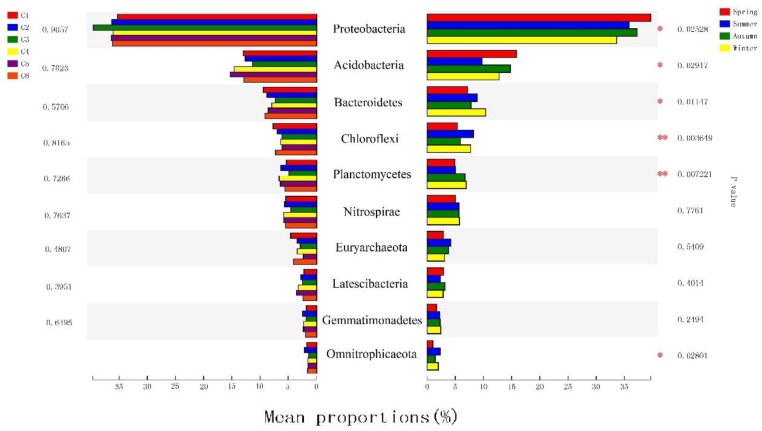
Significantly different relative abundances among seasons and point groups. One-way analysis of variance (ANOVA) was used to evaluate the importance of comparisons between indicated groups. * P < 0.05, ** P < 0.01.

These dominant groups exhibited significant differences among seasons (Figure 4), but there seemed to be no regularity between dominant species and numbers for the different seasons and sites. The most prevalent phylum among all six sample sites, regardless of season, was Proteobacteria. This phylum represented 36.30–39.04% of the microbial community in sediment samples obtained in summer, 37.62–49.06% of that obtained in autumn, 33.20–39.15% of that obtained in winter, and 37.23–45.03% of that obtained in spring (Figure S2A). Unlike previous studies [52,53], Acidobacteria, which exists in the sediment, was the second largest bacterial group in the 24 water samples (summer: 5.69–12.55%; autumn: 10.53–17.32%; winter: 8.21–18.09%; spring: 14.06–18.95%). Notably, CMS3 differed greatly from the other samples, and *Firmicutes* (28.48%) did not appear in the other samples. The bacterial community composition in Lake Chaohu was similar to that of other lake ecosystems that have been studied by high-throughput sequencing. This further suggests that there is indeed a view of “typical freshwater bacterial”, as previously observed in other freshwater lakes [54,55].

A large proportion of sequences were not assigned to any genera. Some of these sequences, such as Unclassified Sinobacteraceae, Unclassified SC-I-84 and Unclassified MBNT15, appeared in all samples but were more abundant in some of the samples than in others (Figure S2B). The abundance of Unclassified Sinobacteraceae in autumn and spring was 7.44–16.03% and 4.69–11.53%, respectively; however, its abundance was only 3.45–6.25% and 3.83–9.67% in summer and winter, respectively. Unclassified MBNT15 was abundant in spring (4.68–7.35%) but much less abundant in the other seasons (summer: 1.61–4.69%; autumn: 2.13–3.89%; winter: 1.20–4.44%).

**Figure 5:**
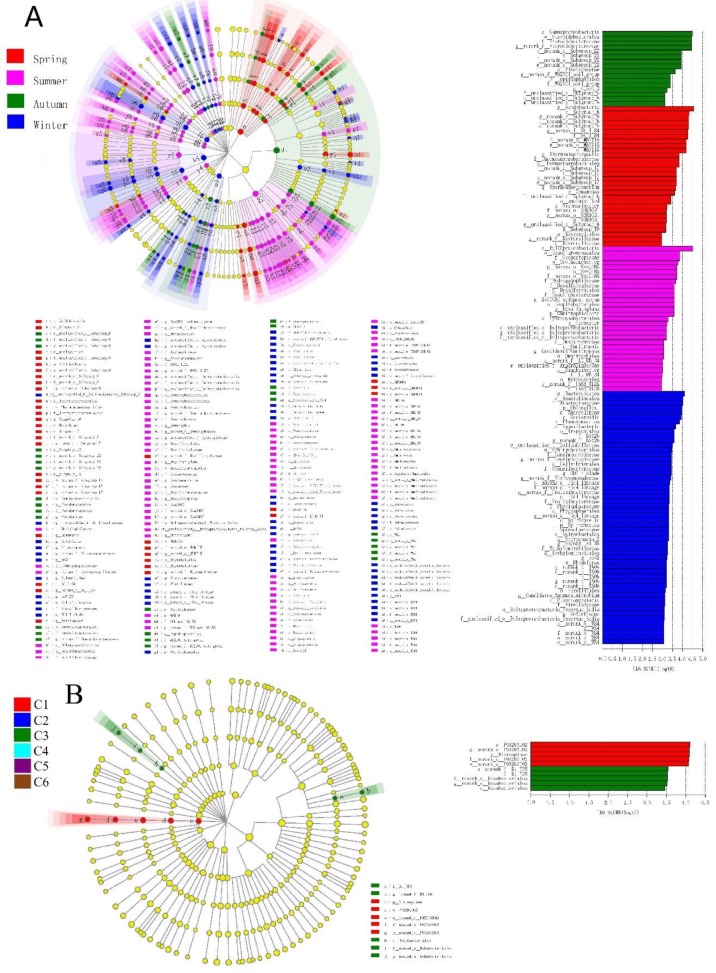
(**A**) Cladogram showing the phylogenetic distribution of the bacterial lineages associated with sediment for the 24 samples in different seasons and locations. The phylum, class, order, family, and genus levels are listed in order from the inside to the outside of the cladogram, and are used to determine the most likely to explain the difference between taxa groups or classified terrain types. (**B**) Indicator bacteria with linear discriminant analysis (LDA) scores of 2 or greater in bacterial communities associated with sediments that differed among seasons and points. Different-colored regions represent different constituents, whereas the yellow circles represent taxa with no significant differences in the sediments.

Groups were represented by a cladogram, and LDA scores of 2 or greater were confirmed by LEfSe analysis (Figure 5B). The cladogram revealed that 8 phyla, 16 classes, 31 orders, 34 families, and 42 genera showed significant seasonal variation (Figure 5A) (Table 2). Many groups of bacteria were significantly enriched. For example, Acidobacteria (from phylum to genus) was prominent in the spring, Verrucomicrobia and Actinobacteria (from phylum to genus) were significantly enriched in summer, and Planctomycetes (from phylum to genus) was enriched in winter. Among the groups that showed the strongest seasonal changes, most had the highest abundance in winter (Table 2). However, in the spatial groupings, fewer microbes were significantly enriched: Nitrospinae (from phylum to genus), Rokubacteriales (from order to genus) and B1 7BS (the family and its genus).

**Figure 6:**
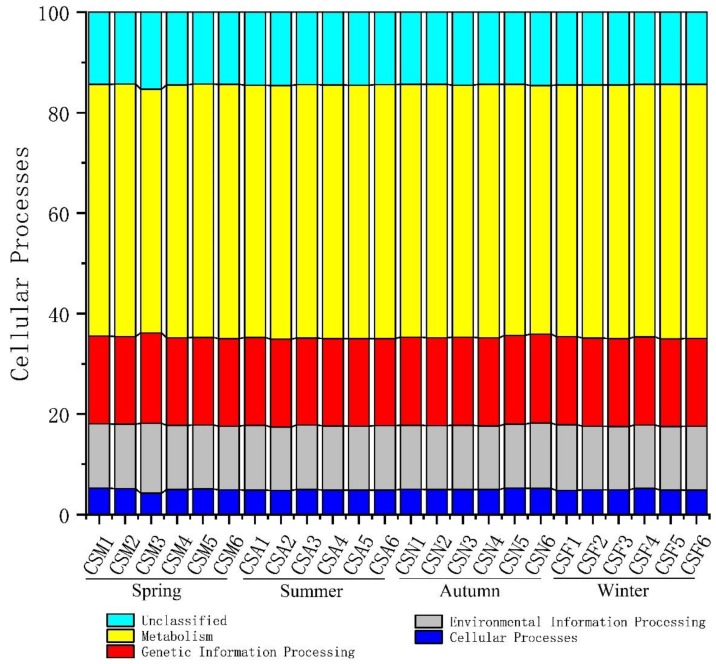
Sampling location and time interval effects on predicted functional groups of OTUs in Lake Chaohu sediment bacteria based on the KEGG (Kyoto Encyclopedia of Genes and Genomes) database. Functional groups with less than 1% relative abundance are not included.

Although we found changes in the bacterial community among seasons and sites, no significant differences in their functional spectra were observed. Analysis of the community KO profiles demonstrated no significant differences in functional groups, regardless of sample location or time interval (Figure 6). Most functional genes were involved in metabolic function (~49.5%), followed by genetic information processing (~17%).

### 3.4. Physical and Chemical Indicators Correlated with Bacterial Community Composition

Redundancy analysis aims to reveal the effects of physical and chemical factors on bacterial community changes in different seasons and regions (Figure S3). Although RDA indicated that the bacterial communities in sediments were weakly correlated with environmental factors, pH was revealed to be the most important factor. In addition, T, TN and TP also affected the construction of bacterial communities in the sediments (Figure S3).

### 3.5. Co-Occurrence Network Analysis

Among the 24 samples from the six sites in Lake Chaohu, co-occurrence network analysis revealed sparse correlations between 50 genera and identified 44 nodes and 218 edges. The average shortest path length was 2.706 edges, with a diameter of 7 edges.

**Figure 7:**
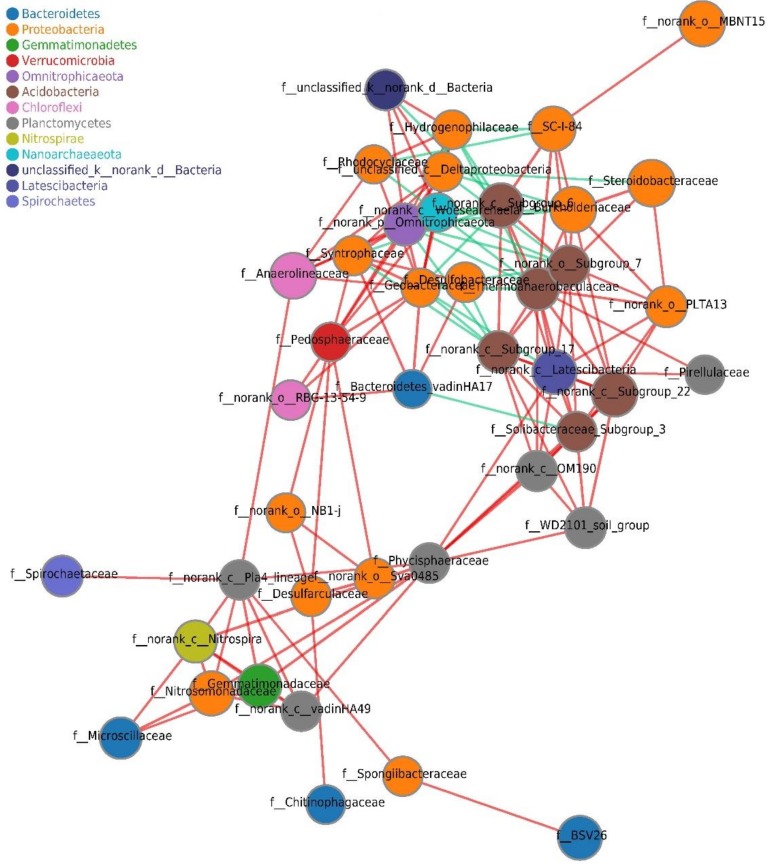
Co-occurrence network analysis applied to the 24 samples at the bacterial family level in Lake Chaohu. Co-occurring network colored by phylum. A connection represents a strong (Spearman’s p > 0.6) and significant (P < 0.05) correlation. The size of each node is proportional to the relative abundance; the thickness of each connection between two nodes (edge) is proportional to the value of Spearman’s correlation coefficients. Red and cyan lines indicate positive and negative correlations, respectively.

Our results indicated that the level of connectivity within the Lake Chaohu bacterial community was high. The nodes in the network were assigned to 13 phyla, 3 of which (Proteobacteria, Planctomycetes, and Acidobacteria) were widely distributed (Figure 7), and the first few families identified as key groups between central scores were Pedosphaeraceae, Phycisphaeraceae, Anaerolineaceae, and Geobacteraceae.

## 4. Discussion

A growing body of research based on high-throughput sequencing technology has revealed the diversity of bacterial communities that live in aquatic environments [56,57,58]. However, most previous studies on the diversity and variability of lake bacterial communities have focused on one aspect, i.e., either a long-term time series or small-spatial-scale environmental gradient [59,60]. Here, we investigated the spatiotemporal patterns of bacterial communities covering the entire lake in spring, summer, autumn and winter. We demonstrated that abiotic factors shaped local microbial community characteristics and might lead to unique bacterial co-occurrence patterns.

The impact of urban emissions on the TOC content was evident in the western part of Lake Chaohu (Table S2). According to previous studies, water with a high organic content may increase or decrease the richness of sediment bacteria [61]. However, the bacterial community exposed to more urban drainage in Lake Chaohu did not differ significantly from the bacterial communities exposed to less drainage at the other sampling sites, which is contrary to the results of other studies. This discrepancy may mean that most of the sediments in Lake Chaohu can utilize various nutrients in the system and/or withstand environmental fluctuations [62,63]. Alternatively, this could be due to most of the studied communities being dormant because they are resistant to severe environmental fluctuations, as studies have shown that dormant cells interfere with community richness [64].

Our study examined the microbial community structure in lake sediments among sites in Lake Chaohu. The dominant group in the community consisted of Proteobacteria (Gammaproteobacteria, Deltaproteobacteria). Members of these two classes have been observed in organic lake sediments [10,65,66] and are considered to be the most abundant groups in eutrophic lakes [67]. Moreover, Gammaproteobacteria has been negatively correlated with NH_4_^+^-N [68], and Deltaproteobacteria was reported to be associated with NO_3_-N and TN [69,70]. Therefore, these classes have strong correlations with the conversion of nitrogen. The dominance of these two groups at each site likely indicates an increase in pollution caused by agricultural products in Lake Chaohu, as there is a large amount of agricultural product pollution in this lake [71,72] and a large amount of nitrogen is present in agricultural products [73]. Moreover, these taxa can be used as reference indicators for agricultural non-point source pollution in the Nanfei River, Zhaohe River, and Tongyang River because sewage from upstream farmland flows into the lake from each river.

Other enriched phyla, such as Acidobacteria and Bacteroidetes, also exhibit good characteristics in sediments. Bacteroidetes are increasingly recognized as effective degraders of high-molecular-weight organic matter, including various enzymes and carbohydrates [74,75], in different ecological niches [76,77]. The partial taxonomic group of Acidobacteria also has the ability to degrade a variety of simple carbon compounds [78] and petroleum compounds [79]. The enrichment of these two groups at various sites suggested that the whole lake was contaminated by organic matter.

The sample from the Zhaohe River estuary in May (CSM3) was different from the other samples. Firmicutes, which were found in only this sample, are usually found in rivers, lakes and glaciers [80,81,82]. Their high enrichment may be due to the impact of water-transfer projects. Due to the eutrophication of Lake Chaohu, policymakers decided to introduce Yangtze River water during the prebloom stage of Cyanobacteria to clean the lake, and the Zhaohe River was used as an intermediary [83]. Some investigations have confirmed that the Yangtze River-Lake Chaohu water transfer project has been conducive to accelerating water flow, enhancing self-purification and alleviating eutrophication in the lake [84]. Although sediment scouring in this lake occurs over a short period of time, it may be severe enough to cause the habitat to be unfavorable for the original microorganisms. The Firmicutes that suddenly appear may come from an exogenous environment because we observed high enrichment of Firmicutes in the Yangtze River [85,86]. Alternatively, regarding functional and ecological characteristics at the family level, Streptococcaceae, which belong to the phylum Firmicutes, are facultatively anaerobic chemo-organotrophs [87], and most of them are pathogenic to humans and other animals [88]. Members of this family are best known for their influence on starter cultures in the yogurt industry [89]. Enterobacteriaceae are ubiquitous in nature, and many are significant human, animal, and/or plant pathogens causing a range of infections. Members of this family can be used as anticancer agents for controlling infectious diseases [90]. Therefore, the community differences may be due to the presence of food factories or large hospitals on the Zhaohe River, as emissions from these areas are brought into the estuary due to water-transfer projects.

As we hypothesized, the abiotic environmental factors and exogenous inputs at different sites in the lake shaped the complex microbial community composition. These results are consistent with previous results and reflect the community’s plasticity and tolerance to the environment [91,92]. However, it should be noted that more information is needed to explore complex patterns of co-occurrence between microorganisms, which are difficult to observe.

Network analysis has been used in microbial ecological studies to visualize the common correlations of bacterial genera and to predict possible ecological processes regulated by bacterial communities [93]. An examination of the topological properties of the network provided valuable supplementary information for the analysis of lake community diversity. The most abundant phyla studied in the co-occurrence network were Proteobacteria, Bacteroidetes and Acidobacteria, which were also dominant taxa in terms of community composition. Pedosphaeraceae, Phycisphaeraceae, Anaerolineaceae, and Geobacteraceae had the top betweenness centrality scores in this study. The hallmark of the genera in family Geobacteraceae is their ability to reduce insoluble Fe and Mn, for which they can employ several mechanisms, most notably extracellular electron transfer via electric conductive nanowires [94]. Although the ecological functions of other dominant species are not clear, previous network analysis makes it easy to speculate that they play an important role in ecological function [29,95].

Functional predictions can reproduce the main findings of the microbial analysis and facilitate metagenomic predictions in a wide range of host-related and environmental samples [31]. Although we found changes in the bacterial community between seasons and sites, no significant differences in functional spectra were observed. This result may have been due to functional interference between the communities [28]. Furthermore, many studies have demonstrated that certain bacteria have strong plasticity [96] and high adaptability [97] and are able to overcome environmental changes to take advantage of previously unavailable resources. For example, one such genus, pseudomonas, encounters an unusual movement selection gradient [98]. In fact, although the functional classification attributes are different, there is a phenomenon in the plant kingdom in which there is no significant relationship between the unique contribution of the environment and the functional traits tested [99]. Alimov et al. [100] reported the stability and complexity of an aquatic animal community in terms of structural and functional characteristics. Therefore, we speculate that environmental control as a determinant of species distributions may be unstable during evolution or that various organisms, including microorganisms, can resist environmental changes and maintain their structure and function.

Alternatively, a large part of the group in the community could be dormant, which is likely to occur [64,101,102] and provides a good explanation for the indistinguishability of the CSM3 sample’s functional genes from those of other samples. Groups with functionally distinct or complementary genes may switch to dormancy due to insufficient resistance to environmental conditions. It is also possible that most of the bacterial systems in this study are poorly identified [10], such that their taxonomic diversity cannot be accurately reflected in the functional profile, resulting in similarity in the bacterial functional profiles of the lake between samples. Thus, although the microbial community functional profile determined by the PICRUSt algorithm provides an overview of functional potential, its accuracy remains to be determined.

## 5. Conclusions

Although community diversity was generally consistent between sites, different abiotic indicators affected bacterial community composition. In the estuary, during the month when the water transfer project was implemented, the bacterial communities in the sediment demonstrated different assembly patterns, as severe sediment scouring caused changes in the nutrient and hydrological characteristics of the original microbial habitat. However, these changes do not seem to have caused fluctuations in bacterial function, suggesting that all or part of the communities in the lake sediments can better resist interference via versatile physiologies and/or functional redundancy. Does this finding mean that community-level changes in function are subtler in benthic ecosystems than in other ecosystems? This question is worth further research. Moreover, the non-random co-occurrence pattern of bacteria in eutrophic lake sediments obtained by employing network analysis of a large number of lake microbial data sets generated by high-throughput sequencing provided a new perspective on microbial assembly in large contaminated lakes. The next logical step is to study the network model mechanism of community changes over time and/or in the lake.

## Figures and Tables

**Table 1:** The ranks of the dissimilarities within and between groups for seasonal and spatial bacterial communities were estimated by ANOSIM (analysis of similarity statistics).

Method	Groups	Statistic	P value	Permutation Number
ANOSIM	Seasonal	0.319	0.001	999
	Spatial	−0.083	0.929	999

**Table 2:** The phylogenetic distribution of bacterial lineages associated with the 4 seasons of a year.

Taxon	No. of Bacterial Taxa with Significant Seasonal Variation	Total no. of Bacterial Taxa in LEfSe Analysis	No. of Bacterial Taxa Enriched in the Sediment During the 4 Seasons			
			Spring	Summer	Autumn	Winter
Phylum	8	15	1	1	0	6
Class	16	32	3	2	3	8
Order	31	65	7	8	4	12
Family	34	78	8	9	4	13
Genus	42	64	101	11	5	15

Note: LEfSe analysis, the LDA Effect Size analysis, is an analytical tool for discovering and interpreting high-dimensional data biomarkers (genes, pathways, taxonomies, etc.) that can be compared between two or more groups.

## Supplementary Materials

The following are available online at https://www.mdpi.com/1660-4601/16/20/3966/s1. Table S1. Estimates of richness and diversity for operational taxonomic units (OTUs) definition of 97% similarity for the four seasons samples obtained from six sampling points in Lake Chaohu. Table S2. The physical and chemical indices of sediment samples in different positions in Lake Chaohu. Figure S1. Rarefaction curves of OTUs clustered at 97% sequence identity across the five samples. Figure S2. Relative abundance of the predominant phyla (A) and genus (B) of each sediment sample.
