# Supplementary material for: Spatial-Temporal Variation of Bacterial Communities in Sediments in Lake Chaohu, a Large, Shallow Eutrophic Lake in China
Source: Int J Environ Res Public Health. 2019 Oct 17;16(20):3966. doi: 10.3390/ijerph16203966 (PMC6844080; doi:10.3390/ijerph16203966)
Supplement: Supplementary file 1 [file ijerph-16-03966-s001.pdf]

Table S1. Estimates of richness and diversity for operational taxonomic units (OTUs) definition of 97% similarity for the four seasons samples obtained from six sampling points in Lake Chaohu.

Sample	Seasonal	Shannon	Simpson	Good's Coverage	OTUs
CSM1	Spring	6.158688	0.010716	0.963511	1492
CSM2		6.279109	0.009461	0.961994	1532
CSM3		4.195906	0.102589	0.979820	1259
CSM4		6.545614	0.006907	0.958524	1604
CSM5		6.376466	0.007003	0.952598	1527
CSM6		6.782754	0.003998	0.954446	1644
CSA1	Summer	6.901564	0.003612	0.977746	1419
CSA2		6.940787	0.004358	0.974198	1613
CSA3		6.805816	0.004791	0.965647	1656
CSA4		6.890361	0.004280	0.971381	1616
CSA5		7.006747	0.003335	0.967587	1676
CSA6		6.928289	0.003716	0.965810	1685
CSN1	Autumn	6.617131	0.004971	0.952344	1599
CSN2		6.621193	0.004677	0.948422	1578
CSN3		6.581582	0.006034	0.950740	1616
CSN4		6.599031	0.004677	0.952337	1597
CSN5		6.405505	0.008451	0.963908	1540
CSN6		6.036937	0.01581	0.965964	1364
CSF1	Winter	6.706535	0.004559	0.961459	1427
CSF2		6.876148	0.003323	0.954703	1655
CSF3		6.845468	0.003560	0.951512	1660
CSF4		6.354485	0.007006	0.961727	1535
CSF5		6.820350	0.003123	0.954349	1641
CSF6		6.865831	0.003100	0.944493	1673

Table S2. The physical and chemical indices of sediment samples in different positions in Lake Chaohu.

Season	Samples	T (℃)	pH	DO (mg/L)	TP (mg/g)	TN (mg/g)	TOC (mg/g)
Summer	CSA1	32.3	8.22	7.49	1.35 ± 0.79	1.42 ± 0.89	12.41 ± 5.7
	CSA2	34.5	8.07	9.33	0.65 ± 0.11	1.58 ± 0.07	10.66 ± 0.67
	CSA3	32.5	8.05	8.09	0.17 ± 0.06	1.31 ± 0.08	3.94 ± 0.13
	CSA4	32.5	8.08	8.29	0.38 ± 0.02	1.67 ± 0.11	4.05 ± 0.32
	CSA5	32.3	8.10	7.81	0.48 ± 0.05	2.07 ± 0.18	4.18 ± 0.17
	CSA6	31.9	8.12	6.79	0.55 ± 0.10	1.39 ± 0.03	4.03 ± 0.15
Autumn	CSN1	15.1	8.34	11.30	1.43 ± 0.60	2.15 ± 1.01	9.83 ± 3.10
	CSN2	16.1	8.01	9.22	0.71 ± 0.14	2.22 ± 0.21	8.82 ± 0.77
	CSN3	14.3	8.25	10.20	0.41 ± 0.08	1.44 ± 0.09	3.07 ± 0.21
	CSN4	14.3	8.24	11.27	0.53 ± 0.09	2.01 ± 0.24	3.23 ± 0.35
	CSN5	15.1	8.22	10.20	0.63 ± 0.11	2.44 ± 0.24	3.61 ± 0.47
	CSN6	15.2	8.01	9.88	0.63 ± 0.09	1.22 ± 0.10	3.97 ± 0.38
Winter	CSF1	2.8	8.49	11.95	0.97 ± 0.37	1.62 ± 0.44	8.62 ± 2.90
	CSF2	2.8	8.28	11.80	0.61 ± 0.12	2.67 ± 0.21	7.24 ± 0.54
	CSF3	2.8	8.37	11.40	0.22 ± 0.09	1.29 ± 0.06	1.49 ± 0.10
	CSF4	2.8	8.36	10.58	0.39 ± 0.09	1.63 ± 0.09	2.16 ± 0.21
	CSF5	2.8	8.36	11.50	0.49 ± 0.14	1.93 ± 0.10	3.07 ± 0.37
	CSF6	3.1	8.26	10.70	0.57 ± 0.11	1.43 ± 0.05	4.61 ± 0.17
Spring	CSM1	20.1	7.68	9.31	1.56 ± 0.71	1.86 ± 0.80	10.53 ± 4.17
	CSM2	24.3	8.04	9.16	0.58 ± 0.07	1.75 ± 0.07	9.55 ± 0.95
	CSM3	20.6	7.75	8.52	0.21 ± 0.03	1.52 ± 0.07	2.75 ± 0.16
	CSM4	20.6	7.66	9.13	0.41 ± 0.07	1.98 ± 0.14	2.98 ± 0.44
	CSM5	20.1	7.57	8.46	0.51 ± 0.10	2.38 ± 0.31	3.89 ± 0.84
	CSM6	20.9	7.88	8.46	0.49 ± 0.05	1.99 ± 0.11	5.72 ± 0.94

Abbreviations: DO, Dissolved oxygen; TP, Total phosphorus; TN, Total nitrogen; TOC, Total organic carbon.

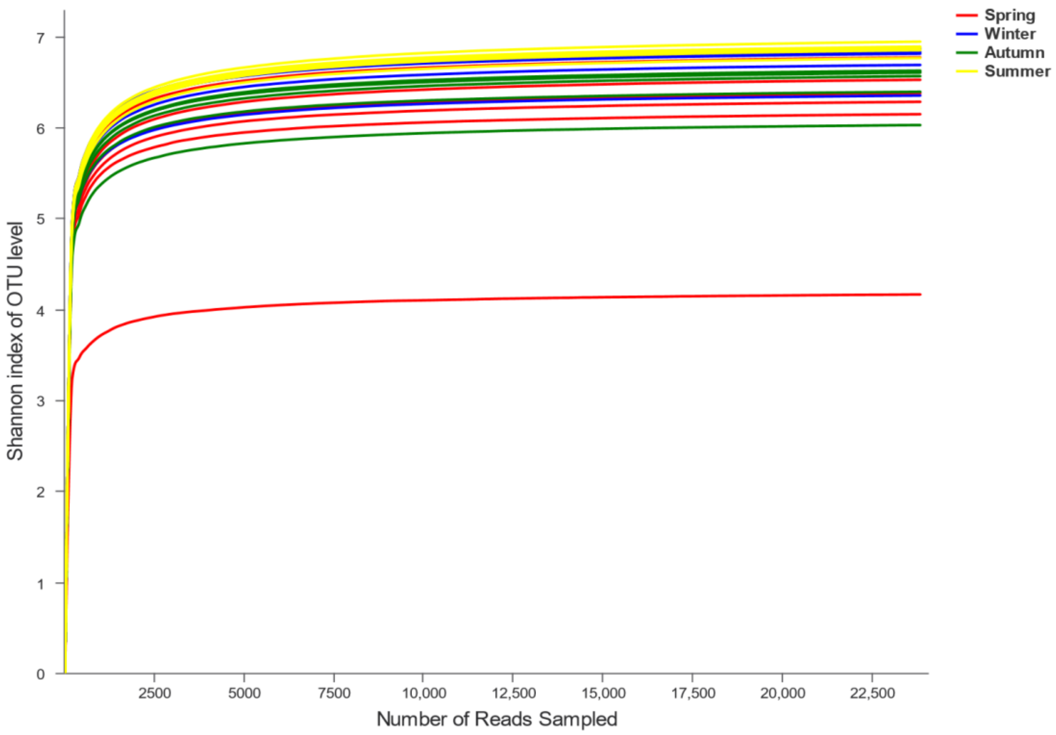

Figure S1. Rarefaction curves of OTUs clustered at 97% sequence identity across the five samples.

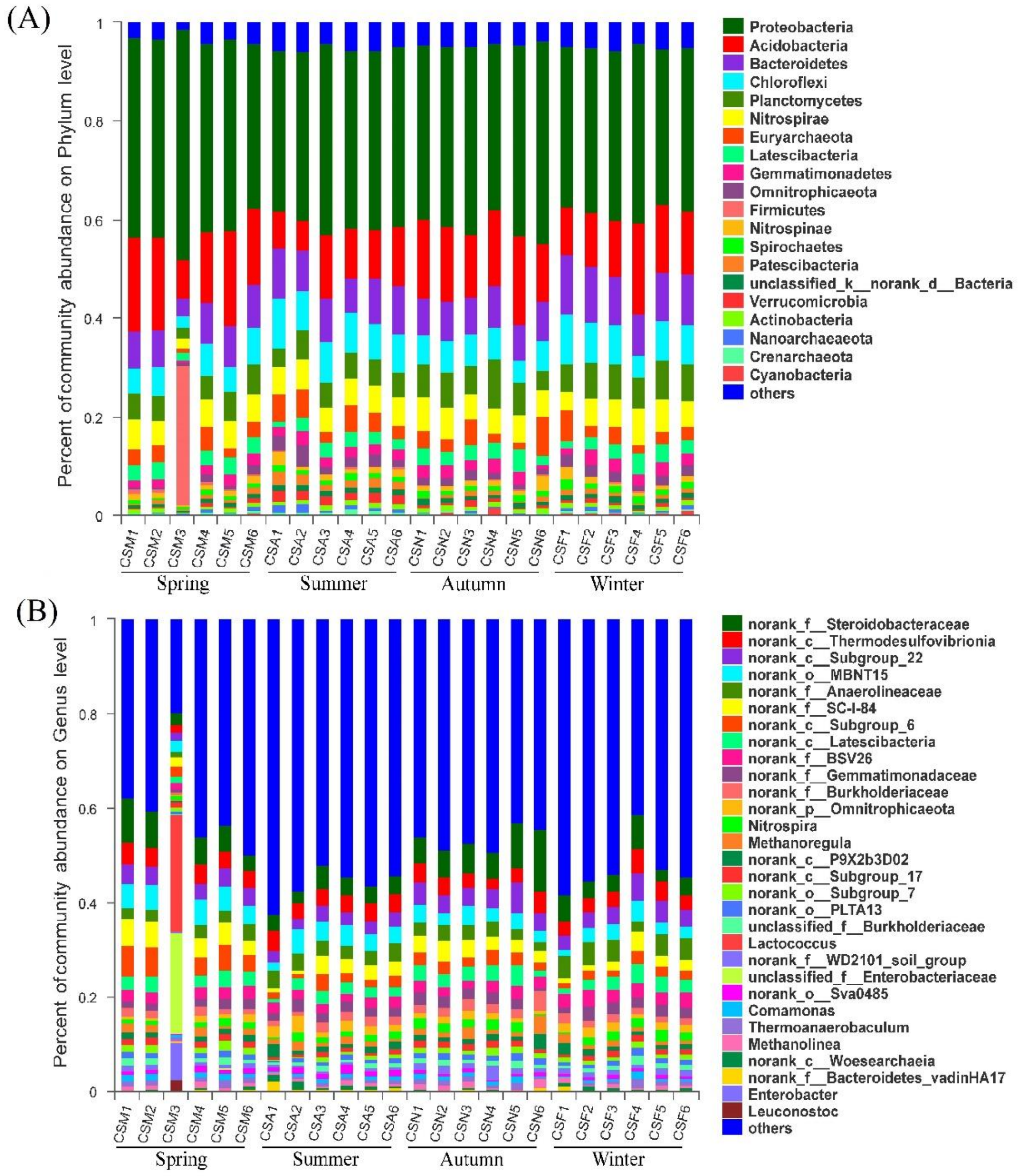

Figure S2. Relative abundance of the predominant phyla **(A)** and genus **(B)** of each sediment sample.

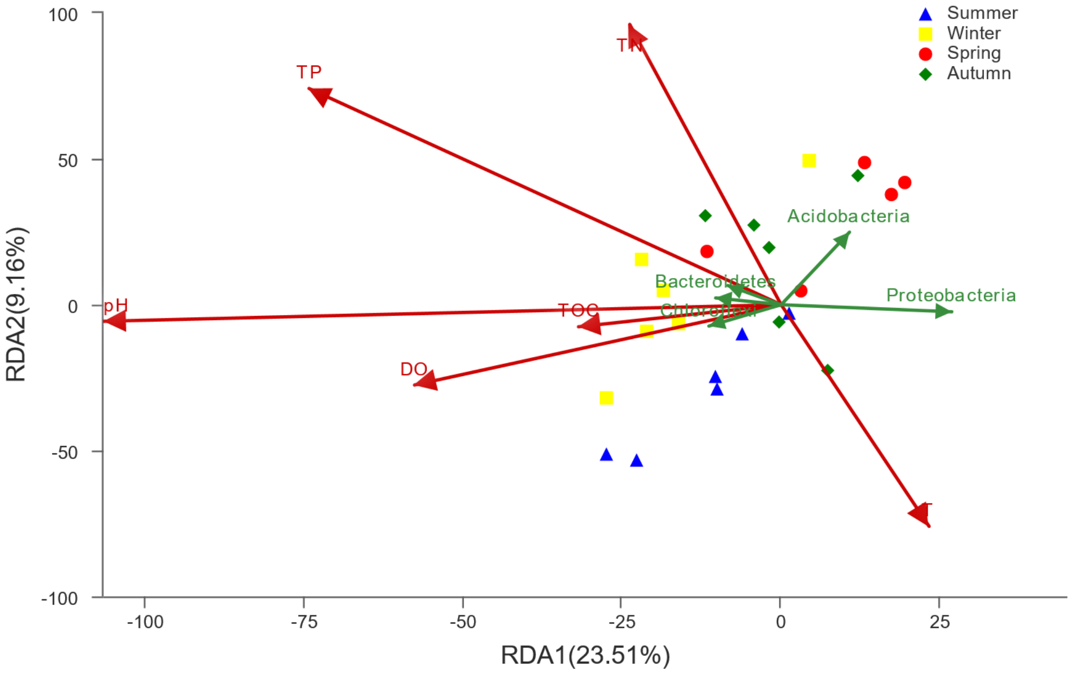

Figure 3. Redundancy analysis (RDA) aims to reveal the effects of physical and chemical factors on bacterial community changes in different seasons and regions, RDA plot for different sampling seasons have an effect on the phylum level of Lake Chaohu (Site 1–6). Arrows indicate the direction of the environmental gradient, and their lengths are proportional to their correlations with the ordination.
